# Rfam 13.0: shifting to a genome-centric resource for non-coding RNA families

**DOI:** 10.1093/nar/gkx1038

**Published:** 2017-11-03

**Authors:** Ioanna Kalvari, Joanna Argasinska, Natalia Quinones-Olvera, Eric P Nawrocki, Elena Rivas, Sean R Eddy, Alex Bateman, Robert D Finn, Anton I Petrov

**Affiliations:** European Molecular Biology Laboratory, European Bioinformatics Institute, Wellcome Genome Campus, Hinxton, Cambridge CB10 1SD, UK; Systems Biology Graduate Program, Harvard University, Cambridge, MA 02138, USA; National Center for Biotechnology Information; National Institutes of Health; Department of Health and Human Services; Bethesda, MD 20894, USA; Department of Molecular and Cellular Biology, Harvard University, Cambridge, MA 02138, USA; Howard Hughes Medical Institute, Harvard University, 16 Divinity Avenue, Cambridge, MA 02138, USA

## Abstract

The Rfam database is a collection of RNA families in which each family is represented by a multiple sequence alignment, a consensus secondary structure, and a covariance model. In this paper we introduce Rfam release 13.0, which switches to a new genome-centric approach that annotates a non-redundant set of reference genomes with RNA families. We describe new web interface features including faceted text search and R-scape secondary structure visualizations. We discuss a new literature curation workflow and a pipeline for building families based on RNAcentral. There are 236 new families in release 13.0, bringing the total number of families to 2687. The Rfam website is http://rfam.org.

## INTRODUCTION

Rfam is a database of RNA families in which each family is represented by a multiple sequence alignment, a consensus secondary structure, and a covariance model (CM). Using a combination of manual, literature-based curation and a custom software pipeline, Rfam converts descriptions of RNA families found in research papers into computational models that can be used to annotate RNAs belonging to those families in any DNA sequence. Valuable research outputs that are often locked up in figures and supplementary information files are encapsulated in Rfam entries and made accessible through the Rfam website and other resources that integrate the Rfam data, such as Ensembl (1) and UCSC (2) genome browsers.

The process of building a new Rfam family starts with extracting RNA sequences and, where possible, sequence alignments and secondary structures, from the scientific literature and manually building a **seed alignment**. The alignment includes a representative set of homologous sequences spanning the family's taxonomic distribution and capturing its sequence variation. Rfam uses the Infernal software package (3) to build CMs and perform iterative sequence- and secondary structure-based searches against a sequence database to find other instances of the family. The Rfam curator examines the search results and chooses a bit score cutoff, also known as a gathering threshold, which separates true from false homologs. Newly found homologs that are not represented by the current seed alignment are incorporated into it, a new model is built, and the search is repeated. This iterative process continues until no new homologs of the family are discovered. The curator also adds Gene Ontology (4) and Sequence Ontology (5) terms and updates Wikipedia pages (6) to provide insight into the biological function of the family. As new information becomes available, existing families are reviewed and updated.

**Figure 1.**
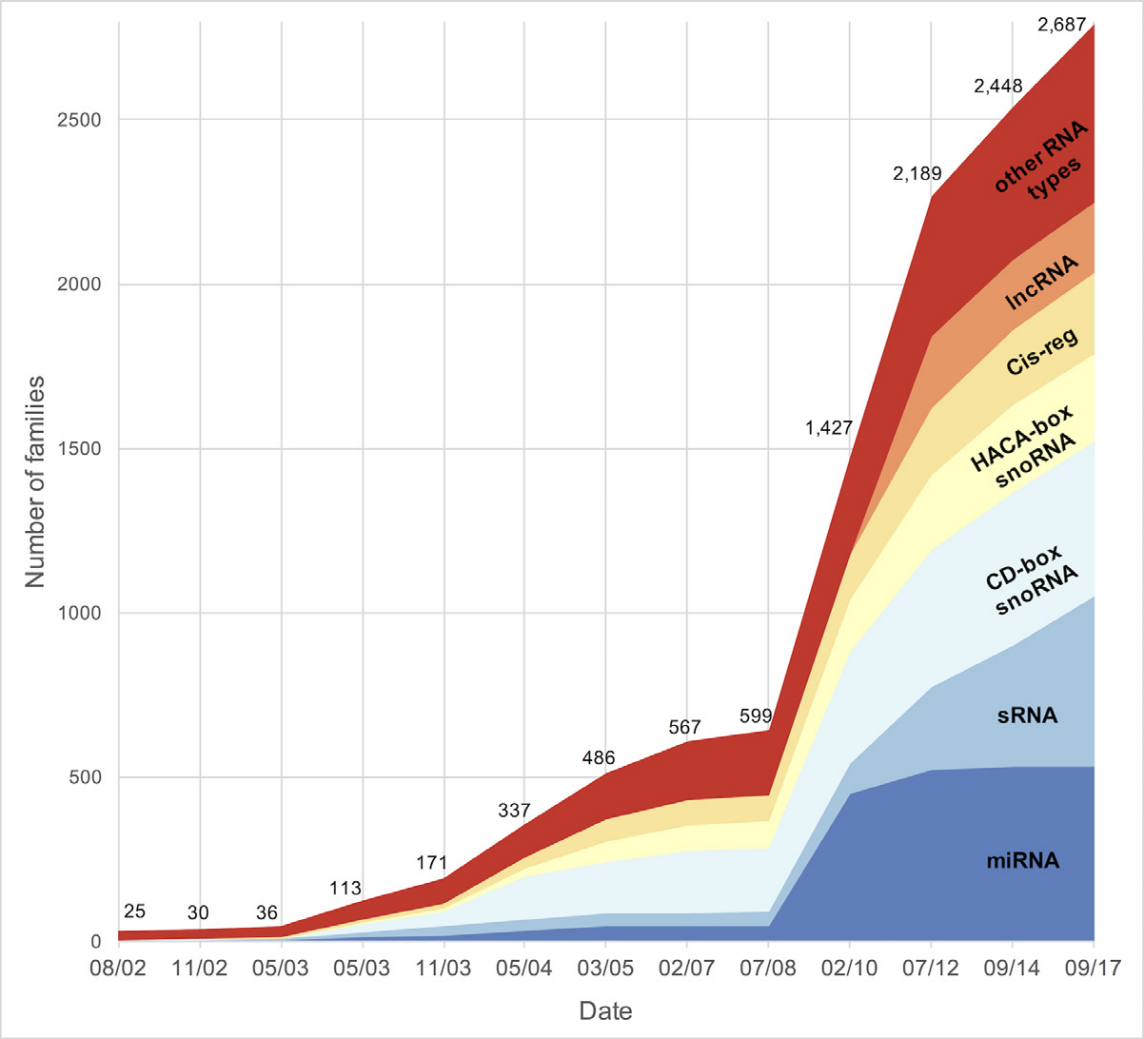
Growth in the number of RNA families grouped by RNA type in major database releases. The *other RNA types* group includes types with less than 50 families, such as rRNA, tRNA, snRNA or riboswitches.

The current release, Rfam 13.0, was made available in September 2017 and contains **2687 RNA families**. Figure 1 shows the growth in the number of RNA families grouped by RNA type in all major releases since 2002 when Rfam was established (7). Some Rfam families are homologous to one another and are only separated to improve annotation because multiple models can be more specific than a single all-encompassing model. For example, there are five small subunit ribosomal RNA families, one each for Archaea, Bacteria, Eukaryota, Microsporidia Fungi and Trypanosomatid mitochondria. Homologous families like these are grouped together into the same **clan** (6). Search results from models in the same clan often overlap so in Rfam we analyse the overlaps and report only the lowest-scoring hits using a procedure called ‘**clan competing**’ (8). In Rfam 13.0, 344 families are organised into 111 clans.

In this article, we discuss the new sequence database composed of complete reference genomes. The shift towards using complete genomes instead of the entire ENA sequence archive significantly reduces data redundancy, enables meaningful taxonomic comparisons, and allows for the ncRNA annotations to be organised and displayed in a genome-specific manner on the Rfam website. We also give an overview of new families and describe a new literature curation workflow.

## UPDATING THE RFAM SEQUENCE DATABASE

To build new families and identify instances of existing ones, Rfam uses an aggregated sequence database called ***Rfamseq***. Previously *Rfamseq* was composed of the standard (STD) and whole genome shotgun (WGS) sequence sets obtained from the ENA (9). Prior to release 13.0 the most recent version of *Rfamseq* was generated based on ENA release 110 (December 2011). Since then the size of the STD and WGS sets has grown almost tenfold from 0.27 Tb to 2.2 Tb (ENA release 132, July 2017), and updating *Rfamseq* with these data would make sequence searches and family building impractical. Furthermore, many of these sequences represent redundant versions of the same or closely related genomes and annotating all of them may not be useful. For example, *Rfamseq* v12 contained 26 Gb of human sequences which is eight times more than the length of reference human genome, and 77 different *Mycobacterium tuberculosis* strains. This redundancy in sequence led to redundant hits in the CM search results and made the analysis of such results difficult.

Sequence redundancy is a common problem for biological databases. To deal with it, the UniProt database recently switched to annotating a non-redundant and regularly updated set of **reference proteomes** which are selected using a semi-automated approach to cover the taxonomic spread of species (10). The reference proteomes are reviewed by UniProt curators who ensure that species of special interest remain in the reference set.

As of Rfam 13.0, we are using the same set of genomes as is used to generate the UniProt reference proteome collection (release March 2017, supplemented by viral genomes used in release January 2017). It includes **8366 genomes** from 8302 different species, with 3025 species in common with *Rfamseq* v12. All genome sequences were downloaded from ENA based on GCA assembly accessions or WGS identifiers obtained from UniProt. The size of the *Rfamseq* v13 database is comparable with the previous version, v12 (Table 1).

**Table 1.** Comparison of *Rfamseq* databases used for Rfam releases 12.3 and 13.0

	*Rfamseq* v12	*Rfamseq* v13
Total sequence length	274 Gb	300 Gb
Number of complete genomes	N/A	8366
Number of species	464 237	8302 (3025 identical with v12)
Number of significant hits	8.7 million	2.3 million

**Table 2.** Summary statistics for Rfam annotation of various genomes

Genome	Assembly/accession	Size	Number of hits	Number of families
*Homo sapiens*	GRCh38.p9	3.2 Gb	14 882	803
*Sus scrofa* (pig)	Sscrofa10.2	2.7 Gb	6289	637
*Drosophila melanogaster*	Release 6 plus ISO1 MT	143.7 Mb	945	160
*Caenorhabditis elegans*	WBcel235	100.3 Mb	1027	175
*Escherichia coli*	U00096.3	4.6 Mb	382	130
*Bacillus subtilis*	ASM904v1	4.2 Mb	250	53
*Methanocaldococcus jannaschii*	ASM9166v1	1.7 Mb	276	18
*Aquifex aeolicus*	ASM862v1	1.6 Mb	55	7
*Borrelia burgdorferi*	ASM868v2	1.5 Mb	42	7

**Figure 2.**
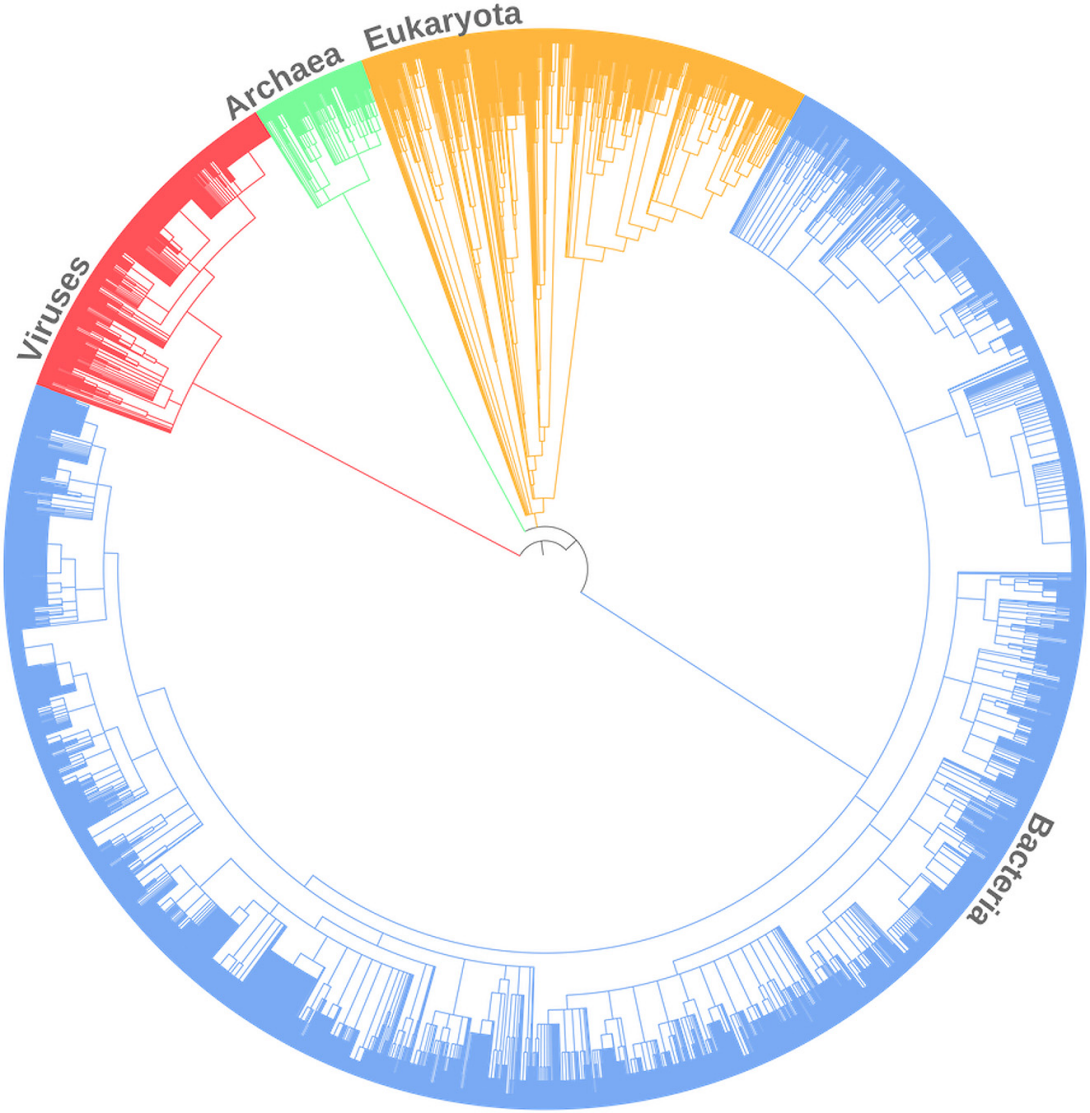
Overview of species annotated with RNA families in Rfam 13.0. The tree is based on NCBI Taxonomy and was generated using iToL (11).

The updated *Rfamseq* has a broad taxonomic coverage shown in Figure 2. The majority of genomes come from Bacteria (∼60%), while Eukaryotes and Archaea account for 10% and 3% respectively. Twenty seven percent of genomes in the database are from Viruses but only 748 out of 2404 viral genomes had Rfam matches.

We searched the new *Rfamseq* with Rfam CMs and found that almost all families had matches to the new genome-centric database. To recover the missing families, we added 26 genomes and viroid sequences that were not present in UniProt. We compared our latest annotations for the genomes that were previously discussed in the Rfam 12 paper (8), and found that the results for the set of common families are comparable between release 12.0 and 13.0 (Table 2). For example, the human genome had matches to 796 families in Rfam 12.0 and 795 in Rfam 13.0. The only missing family was mir-689 (RF00871) that was recently removed from Rfam (see below). It is important to note that not every Rfam hit represents a functional RNA because many hits originate from **pseudogenes** that are no longer active but still maintain enough sequence similarity to be detected as statistically significant hits by CM searches.

In future releases, we will supplement *Rfamseq* with genomes from Ensembl (1), Ensembl Genomes (12) and NCBI reference genome collection (13). We will also consider adding genomes upon user request. In addition, any genome or metagenomic dataset can be annotated with RNA families locally using Infernal and Rfam CMs (for detailed instructions see Rfam Help at http://rfam.readthedocs.org or the Infernal User Manual at http://eddylab.org/infernal/).

## WEBSITE UPDATES

### Text search

Until recently, the Rfam website provided several distinct types of search functionality, such as taxonomy, RNA type and keyword searches. To enable more powerful searches that allow a combination of these search types, we implemented a new **unified text search** that brings all of the website search functionality into one search box. Users can filter the data by RNA type or organism using **facets** and browse genomes, families, clans, motifs, and sequences with rich search results showing key information about each entry.

**Figure 3.**
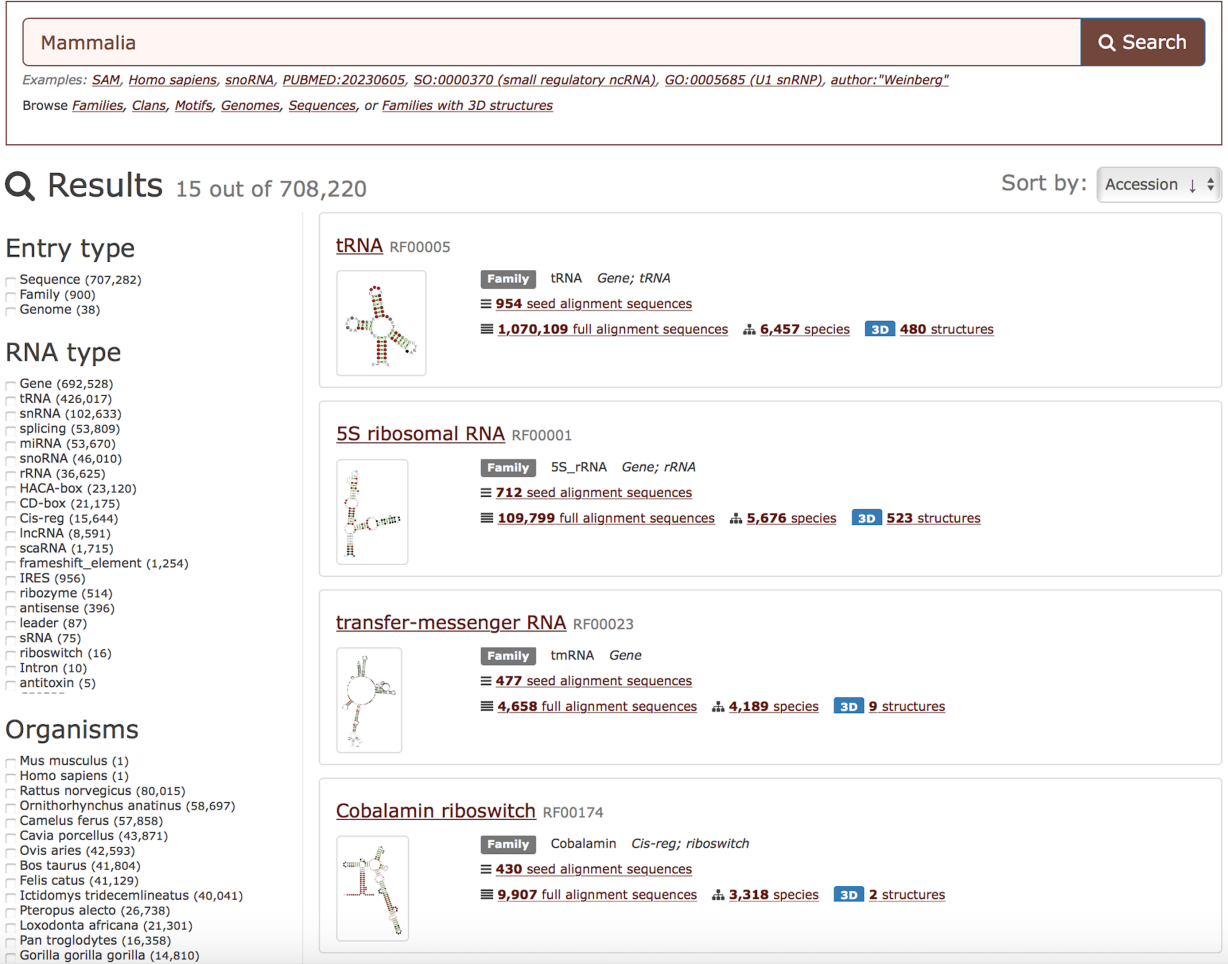
Browsing Mammalian families and genomes annotated in Rfam. The entries can be filtered using facets and sorted by multiple criteria.

Users can search and browse all genomes as well as RNA families and sequences found therein. It is also possible to compare RNA families across taxonomic groups. For example, one can search for *Mammalia* and identify all RNA families found in Mammals (Figure 3). It is also possible to compare any set of genomes to find the families that are common to all of them or specific to some of them. The documentation and examples are available on the Rfam Help website (http://rfam.readthedocs.org). For even more complex queries that are not currently supported in the web interface, users can query the new public MySQL database (see section Availability).

### Sequence summary pages

**Figure 4.**
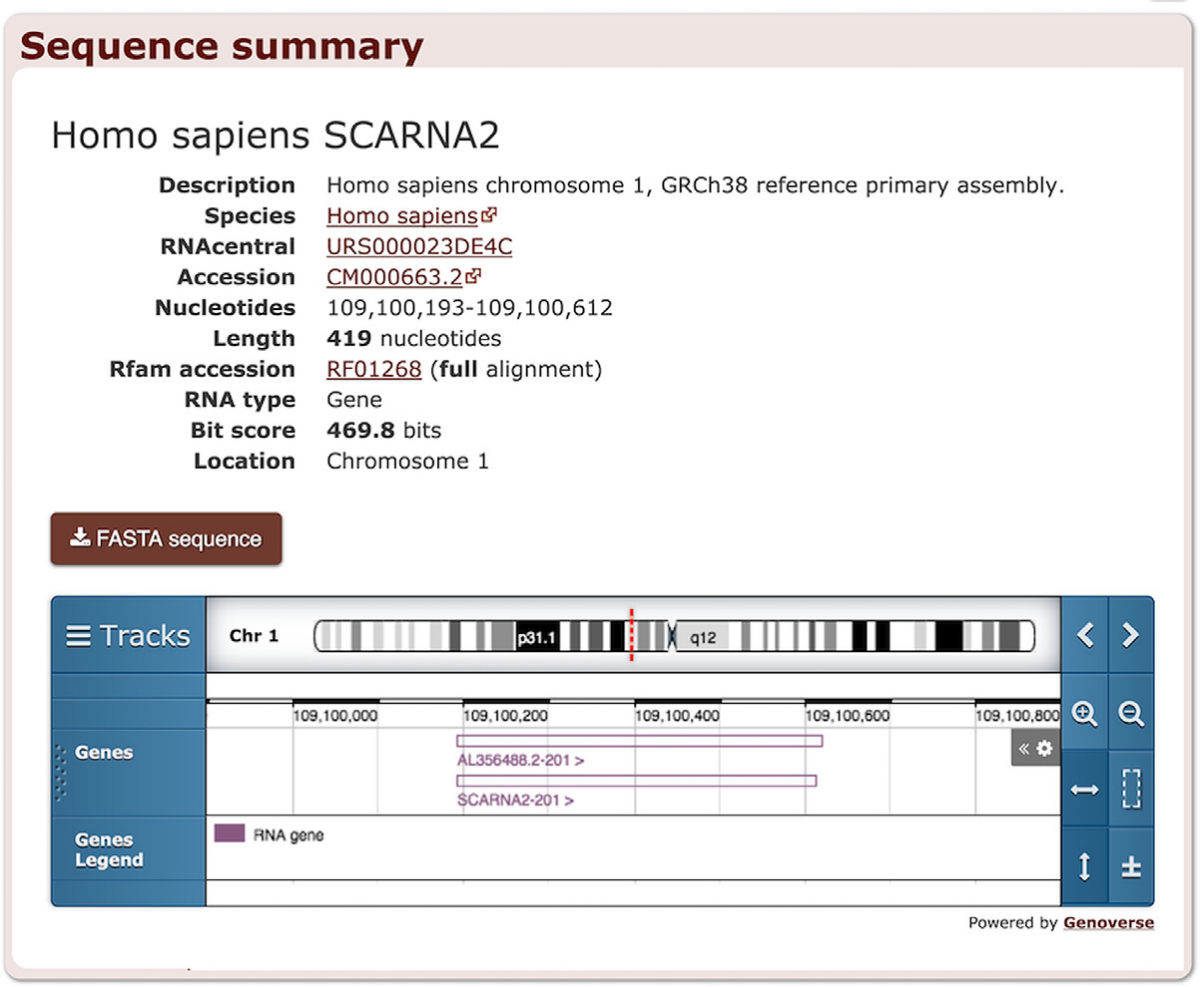
Sequence summary page for Homo sapiens small Cajal body-specific RNA 2 sequence located in chromosome 1.

We also developed new summary pages for each individual sequence that includes a download link as well as links to RNAcentral, ENA, and NCBI Taxonomy. The sequence pages make it possible for other resources to link to Rfam sequences and for Rfam to display more data about each identified RNA, such as its **genomic context** (Figure 4). The pages contain an embedded genome browser powered by Genoverse (http://genoverse.org) that displays genes retrieved using Ensembl API (14).

### R-Scape secondary structures

**Figure 5.**
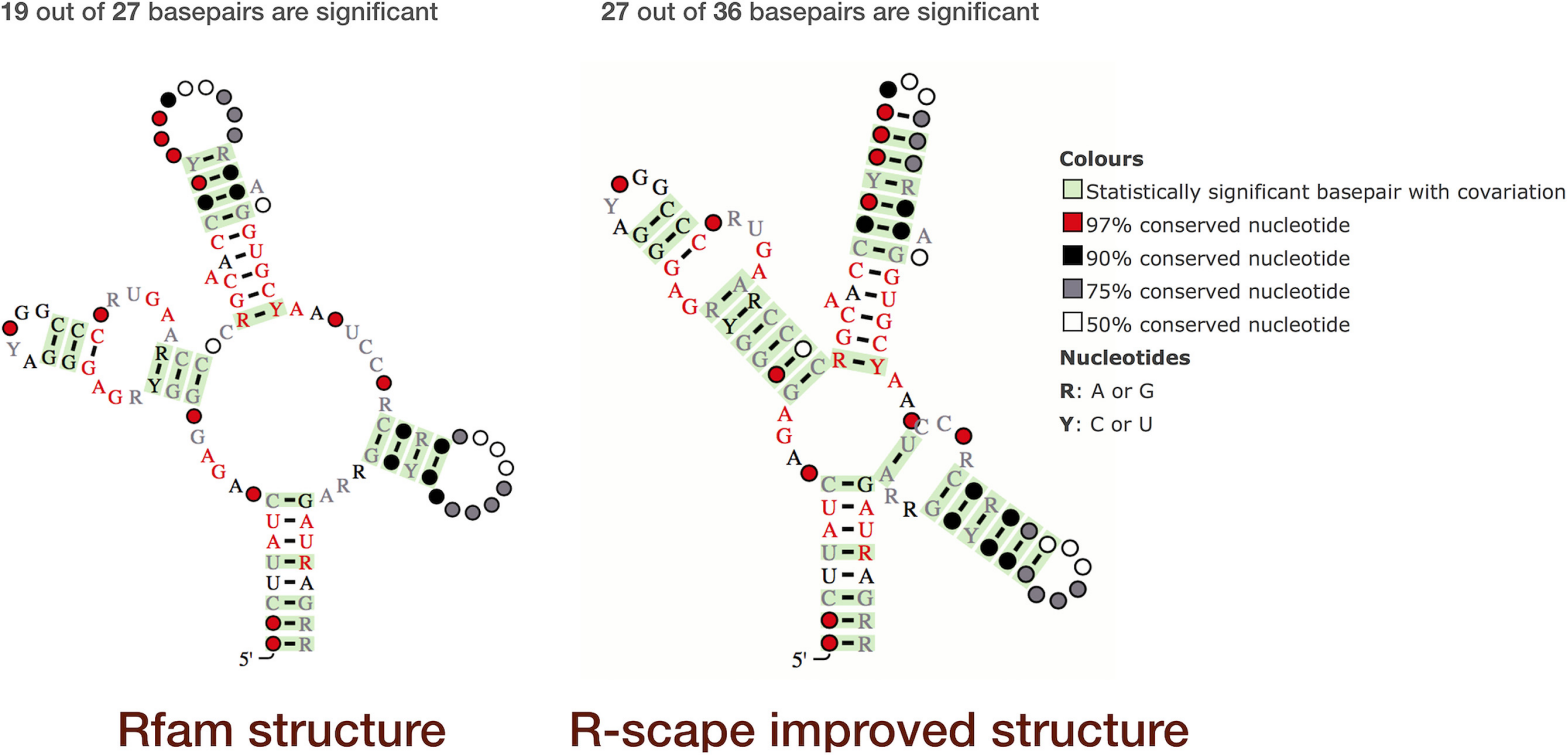
R-scape visualisation of SAM riboswitch (RF00162, http://rfam.org/family/SAM). Left: The current Rfam 13.0 SAM riboswitch seed alignment and consensus secondary structure; 19 of the 27 basepairs in the alignment show statistically significant covariation. Right: The R-scape improved SAM riboswitch seed alignment and consensus secondary structure; 27 out of 36 basepairs show statistically significant covariation. The structures are displayed using R2R (16); significant basepairs, as defined by R-scape, are shown in green. Other colours and markup of the structure diagrams are explained in the legend on the far right.

R-scape (15) is a new method for testing whether covariation analysis supports the presence of a conserved RNA secondary structure. In order to check the quality of Rfam structures, we analysed all Rfam seed alignments with R-scape and added interactive R-scape visualisations to the secondary structure galleries. For example, Figure 5 shows the R-scape analysis of the SAM riboswitch alignment from the Rfam website.

According to R-scape, the secondary structure from the Rfam seed alignment, shown on the left, has 19 statistically significant basepairs (highlighted in green). R-scape can also use statistically significant basepairs as constraints to predict a new secondary structure that is consistent with the seed alignment. Using this approach, R-scape increased the number of statistically significant basepairs from 19 to 27 and added nine new basepairs consistent with the seed alignment (structure on the right). The R-scape secondary structure is also in perfect agreement with that obtained from the crystal structure (17). In this case R-scape indicates that the SAM seed alignment may need to be updated.

R-scape analysis suggests that many existing Rfam secondary structures can be improved (for example, SAM and FMN riboswitches, 5S rRNA). In other families, the current consensus secondary structures are not statistically supported by the R-scape covariation analysis (for example, oxyS RNA) which indicates that either their seed alignments need to be expanded (to increase statistical significance) or that these RNA families do not have a conserved secondary structure. Lastly, there are also cases where the R-scape structures do not show significant improvement compared to the current secondary structure (for instance, Metazoa SRP).

In future releases, we will use R-scape to improve existing Rfam seed alignments and at the time of creating new families. In the meantime, Rfam users can get an indication of the quality of the structure using the R-scape visualisations shown on the website.

## INCREASING THE NUMBER OF RNA FAMILIES

### New literature curation workflow

Extracting sequences from the literature is a time-consuming and error-prone process because the sequences need to be re-typed manually from figures or other images found in papers. To speed up this process, we established a new literature workflow in collaboration with a biocuration company *E-Merge Tech* (http://e-mergeglobal.com). The Rfam team finds papers that potentially contain descriptions of new RNA families (∼15 papers per month on average). The PubMed identifiers of these papers are sent to *E-Merge* curators who extract RNA sequences from text, figures, and supplementary information and capture the metadata associated with sequences, such as experimental evidence, accessions, species, and secondary structure or alignment availability. In addition, *E-Merge* curators search the sequences against Rfam in order to prioritise sequences that do not match any existing Rfam family.

The data is exchanged using a shared Google Spreadsheet which allows us to keep track of the data and perform automatic validation. A copy of the spreadsheet from September 2017 is available at https://goo.gl/hb6nEj and in the Supplementary Information. We also developed an extension for Google Chrome browser (https://github.com/Rfam/rfam-pubmed-id-highlighter) that connects to the spreadsheet and highlights PubMed search results that have not been analysed yet.

**Figure 6.**
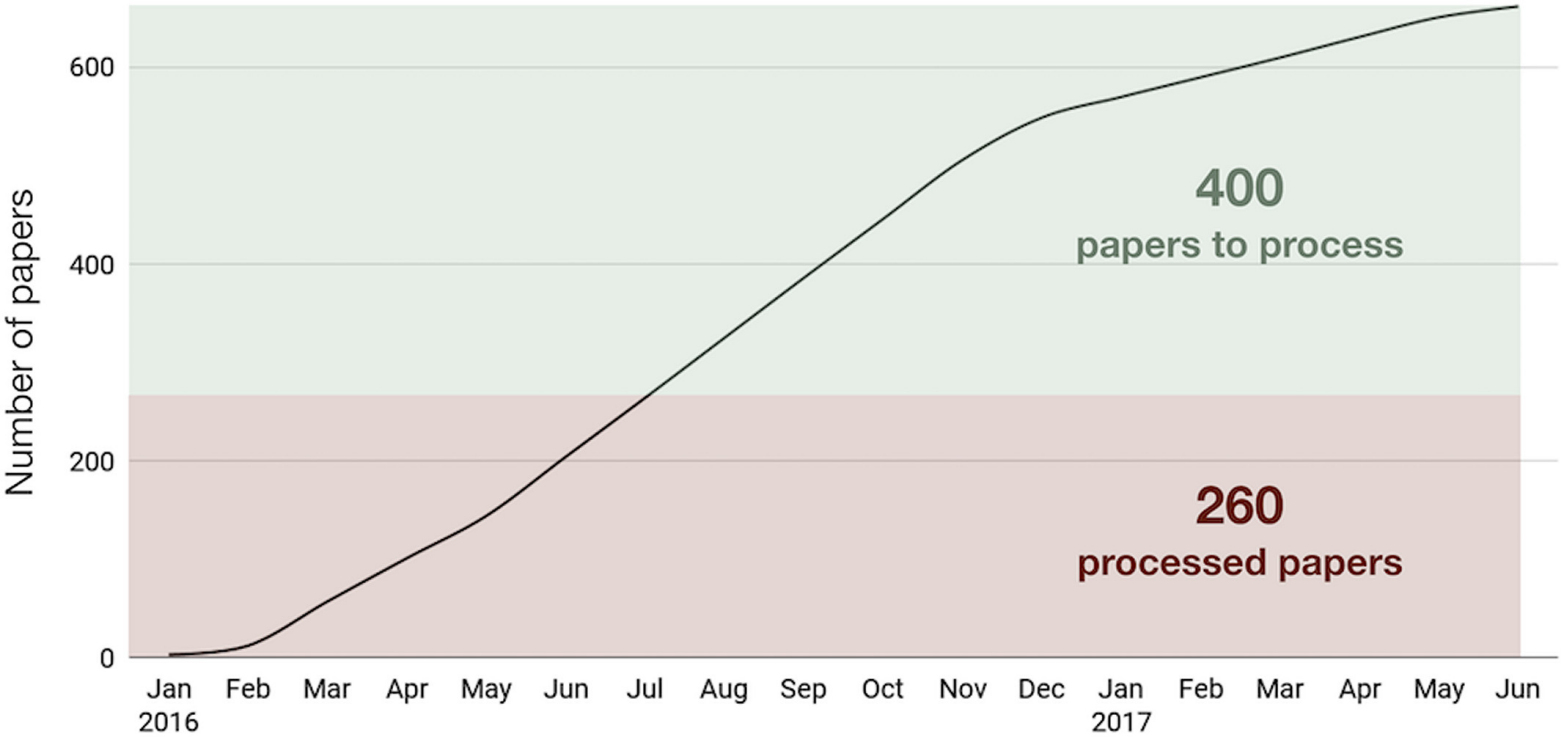
The number of papers curated to extract RNA sequences between January 2016 and June 2017. In total, 660 papers were selected for curation, 260 of which were processed to build 236 new families (some papers were not used for family building for various reasons).

Using this workflow between January 2016 and September 2017 we identified over 660 papers for curation and built 236 families after analysing ∼40% of the papers (Figure 6). If the current trend continues, there may be up to 370 new RNA families that could be added to Rfam based on the already accumulated literature alone.

### Overview of new RNA families

The work described above demonstrates that we are far from having a complete collection of non-coding RNA families in Rfam. Therefore, it is is very important to continue to build new families that we or others have identified. Over the course of three Rfam releases (12.1, 12.2 and 12.3) 236 new RNA families were made available (Table 3). The number of small RNA (sRNA) and thermoregulator families was significantly expanded by the addition of 170 new entries. The group of iron-related bacterial small RNAs was also expanded with HrrF RNA (RF02728) (18) and four Aggregatibacter iron-regulated sRNAs called JA01 to JA04 (RF02729-RF02732) (19). Two new riboswitch families were also added: NiCo (20) and Twister-sister (21).

**Table 3.** Overview of new families added to Rfam since release 12.0

RNA type	Number of families	Example families
Gene; sRNA	148	PsrR1, AsdA, MicL, RnaG, SprX, SorY
Cis-reg; thermoregulator	22	TrxA_thermometer, ToxT_thermometer, AilA_thermometer
Cis-reg	17	Oskar_OES, HilD 3΄UTR, DapZ
Gene; antisense	14	AsrC, Sernc350
Gene; snRNA; snoRNA; CD-box	7	sno_ZL1, sno_ZL2
Gene; snRNA; snoRNA; scaRNA	11	sca_ncR14, sca_ncR26
Gene; ribozyme	5	Hatchet, Pistol, Twister-sister
Gene	3	EBv-sisRNA-1, EBv-sisRNA-2
Cis-reg; riboswitch	2	NiCo, PreQ1-III
Gene; rRNA	2	ppoRNA, hveRNA
Cis-reg; IRES	2	IRES_RhPV, IRES_cyp24a1
Gene; snRNA; snoRNA; HACA-box	3	B_rapa_snoR775, sno_ncR1
	**Total: 236**	

Some of the new RNA families are species-specific including *Francisella tularensis* sRNAs Ftr A (RF02747) and Ftr B (RF02748), which are the first novel small RNAs identified in *F. tularensis* (22).

Nine of the existing families were renamed to reflect new research findings (the details can be found in Supplementary Information Table S1). A new clan (CL00113) was created to group together two 5S rRNA families, 5S_rRNA (RF00001) and mtPerm-5S (RF02547). The 5_8S_rRNA (RF00002) family was added to the LSU clan (CL00112) due to its homology with the 5΄ end of bacterial and archaeal LSU rRNA (23), and *Yersinia* sRNA 186/sR026/CsrC (RF02767) family was added to the Csr-Rsm protein binding clan (CL00106). The RNAIII secondary structure (RF00503) has been updated based on the report by Gupta and colleagues (24).

Three families have been removed since Rfam 12.0. The PK-G12rRNA 23S rRNA pseudoknot family (RF01118) has been deleted because it was covered by the models from the large ribosomal subunit clan. Sau-50 (RF02391) failed protein-coding check, and mir-689 (RF00871) was identified as an rRNA fragment (25) and has been suppressed in miRBase.

### RNAcentral as a source of new families

**Figure 7.**
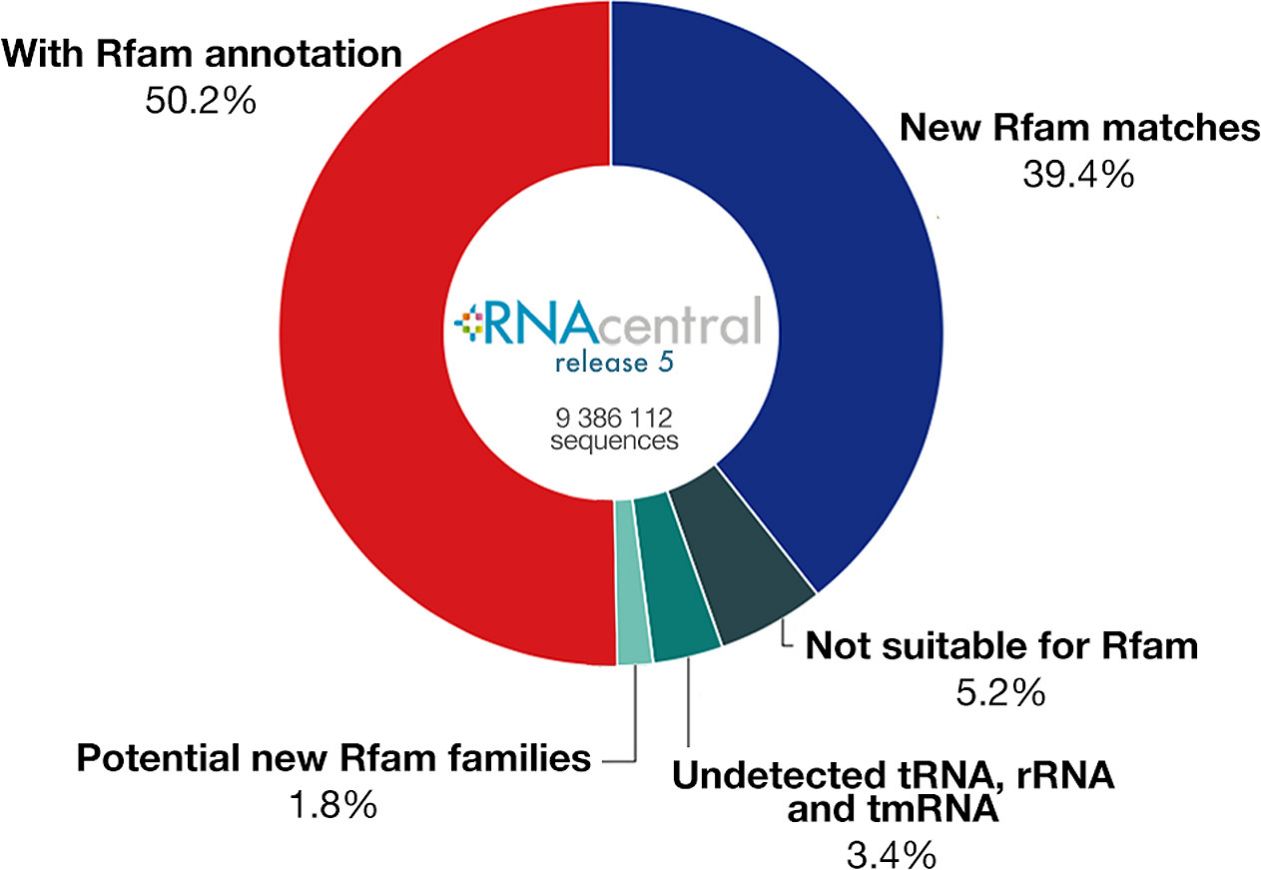
Annotating RNAcentral with Rfam families. About 1.8% of RNAcentral could be used as a source of new Rfam families.

We investigated how well Rfam covers non-coding RNA sequence space by looking at the percentage of RNAcentral (26) sequences that match Rfam families. RNAcentral contains RNA sequences imported from 25 specialised databases, including Rfam, GtRNAdb, Flybase and others. We scanned 9.3 million RNAcentral sequences (release 5) with Rfam CMs and discovered that ∼90% either have been submitted by Rfam (50%) or match an Rfam family (40%). This finding suggests that Rfam already contains the most common RNA families (Figure 7).

The remaining 10% of RNAcentral sequences do not match any existing Rfam family and could potentially represent new families. Some of these sequences belong to non-coding RNA classes that are not suitable for inclusion in Rfam because they are unstructured or their sequence length is too short or too long to be effectively modeled using CMs, such as piRNAs, rasiRNAs, siRNA, and long non-coding RNAs (5% of the total number of sequences).

About 3% of the unmatched sequences are annotated in RNAcentral as being members of ncRNA families for which there is a corresponding Rfam model, yet the existing models do not detect these apparent homologs. These include rRNA, tRNA and tmRNA sequences, and we are investigating these cases to see if the corresponding Rfam models need to be revised or if the RNAcentral annotations are inaccurate.

We focused on the remaining 2% of RNAcentral sequences that could potentially represent new Rfam families. We developed a set of filters based on sequence length and keywords found in sequence descriptions and associated publications to discard sequences that are not suitable for Rfam (mislabelled tRNAs, ITS and others). This narrowed down the list of sequences that may represent potential new families to 41,985 entries. These sequences were then clustered using CD-HIT-EST (27) and the clusters were manually reviewed. We identified several groups of well-described non-coding RNAs and created new Rfam families (EBER-2 and MCS4 sRNAs and snopsi28S-3378 snoRNA). We are currently refining our clustering approach in order to fully automate the identification of candidates for family building.

## AVAILABILITY

The Rfam website is now hosted under its own domain name and is available at a **new URL**, http://rfam.org. We will maintain the redirection from the previously used URL (http://rfam.xfam.org) for the foreseeable future, but users are encouraged to update their links accordingly.

In order to make it easier to query the data in ways that are not supported by the website, we created a **public MySQL database** with the latest Rfam data. Now, users can explore the data using SQL queries from a MySQL client or programmatically using custom scripts. The MySQL database is updated with each Rfam release. Database dumps are archived on the **FTP site** (ftp://ftp.ebi.ac.uk/pub/databases/Rfam) that also contains FASTA files, seed alignments, and CMs for all families.

The Rfam **documentation** has been migrated to a dedicated documentation hosting service, ReadTheDocs, and is available at http://rfam.readthedocs.org. Rfam code and documentation are available on GitHub at http://github.com/Rfam under the Apache 2.0 license. We welcome feedback and questions, which can be directed to rfam-help@ebi.ac.uk or submitted on GitHub.

## CONCLUSIONS

The new Rfam genome-based sequence database is more **scalable** and gives a more accurate view of the distribution of Rfam entries. Using complete genomes enables **meaningful taxonomic comparisons** and identification of a repertoire of RNA families found in a certain species. Users can now more easily and more powerfully search the Rfam data with the new faceted text search. Together these improvements make Rfam a more valuable resource for the sequence analysis community.

## Supplementary Material

Supplementary Data
