# Supplementary material for: Rfam 13.0: shifting to a genome-centric resource for non-coding RNA families
Source: Nucleic Acids Res. 2017 Nov 3;46(Database issue):D335–42. doi: 10.1093/nar/gkx1038 (PMC5753348; doi:10.1093/nar/gkx1038)
Supplement: Supplementary Data [file gkx1038_supp.zip › nar-02837-data-e-2017-File011.pdf]

Supplementary Information Table 1. Changes in Rfam family names and descriptions in release 13.0

**Rfam accession**	**New ID**	**Old ID**	**New Description**	**Reference**
RF01404	PinT	STnc440	PinT (STnc440) Hfq binding RNA	PMID 26789254
RF00111	SdsR_RyeB	RyeB	SdsR_RyeB RNA	PMID 22180532
RF00115	McaS	IS061	McaS/IsrA RNA	PMID 22289118
RF00442	ykkC-yxkD	ykkC-yxkD	Guanidine-I*	PMID 27989440
RF01068	mini-ykkC	mini-ykkC	Guanidine-II*	PMID 28001368
RF01763	ykkC-III	ykkC-III	Guanidine-III*	PMID 28001372
RF01734	Fluoride	CrcB	Fluoride riboswitch	PMID 22194412
RF01750	ZMP_ZTP	Pfl	ZMP/ZTP riboswitch	PMID 25616067
RF01482	AdoCbl riboswitch	Rli55	AdoCbl riboswitch	PMID 25794617

* - only description was updated
